# Predicting preload responsiveness using simultaneous recordings of inferior and superior vena cavae diameters

**DOI:** 10.1186/s13054-014-0473-5

**Published:** 2014-09-05

**Authors:** Hélène Charbonneau, Béatrice Riu, Matthieu Faron, Arnaud Mari, Matt M Kurrek, Jean Ruiz, Thomas Geeraerts, Olivier Fourcade, Michèle Genestal, Stein Silva

**Affiliations:** Département d’Anesthésie Réanimation, CHU Purpan, Université Toulouse 3 Paul Sabatier, Place du Dr Baylac, Toulouse Cedex 9, F-31059 France; EA 4564 “Modélisation de l’agression tissulaire et nociceptive”, University Toulouse 3 Paul Sabatier, Toulouse, France; Department of Biostatistics and Epidemiology, Gustave Roussy, Villejuif, France; Department of Anesthesia, University of Toronto, Toronto, ON Canada; INSERM U825, CHU Purpan, Place du Dr Baylac, Toulouse Cedex 9, F-31059 France; Réanimation Polyvalente et Médicine Hyperbare, CHU Purpan, Université Toulouse 3 Paul Sabatier, Place du Dr Baylac, Toulouse Cedex 9, F-31059 France

## Abstract

**Introduction:**

Echocardiographic indices based on respiratory variations of superior and inferior vena cavae diameters (ΔSVC and ΔIVC, respectively) have been proposed as predictors of fluid responsiveness in mechanically ventilated patients, but they have never been compared simultaneously in the same patient sample. The aim of this study was to compare the predictive value of these echocardiographic indices when concomitantly recorded in mechanically ventilated septic patients.

**Methods:**

Septic shock patients requiring hemodynamic monitoring were prospectively enrolled over a 1-year period in a mixed medical surgical ICU of a university teaching hospital (Toulouse, France). All patients were mechanically ventilated. Predictive indices were obtained by transesophageal and transthoracic echocardiography and were calculated as follows: (Dmax − Dmin)/Dmax for ΔSVC and (Dmax − Dmin)/Dmin for ΔIVC, where Dmax and Dmin are the maximal and minimal diameters of SVC and IVC. Measurements were performed at baseline and after a 7-ml/kg volume expansion using a plasma expander. Patients were separated into responders (increase in cardiac index ≥15%) and nonresponders (increase in cardiac index <15%).

**Results:**

Among 44 included patients, 26 (59%) patients were responders (R). ΔSVC was significantly more accurate than ΔIVC in predicting fluid responsiveness. The areas under the receiver operating characteristic curves for ΔSVC and ΔIVC regarding assessment of fluid responsiveness were significantly different (0.74 (95% confidence interval (CI): 0.59 to 0.88) and 0.43 (95% CI: 0.25 to 0.61), respectively (*P* = 0.012)). No significant correlation between ΔSVC and ΔIVC was found (*r* = 0.005, *P* = 0.98). The best threshold values for discriminating R from NR was 29% for ΔSVC, with 54% sensitivity and 89% specificity, and 21% for ΔIVC, with 38% sensitivity and 61% specificity.

**Conclusions:**

ΔSVC was better than ΔIVC in predicting fluid responsiveness in our cohort. It is worth noting that the sensitivity and specificity values of ΔSVC and ΔIVC for predicting fluid responsiveness were lower than those reported in the literature, highlighting the limits of using these indices in a heterogeneous sample of medical and surgical septic patients.

## Introduction

In patients who present in septic shock, circulatory failure is often the result of hypovolemia, which must be corrected [1]. Volume expansion improves prognosis in this scenario, whereas inappropriate use of vasoconstrictors can lead to harmful tissue hypoperfusion [2]. However, volume expansion may prove ineffective or even deleterious through worsening of preexisting heart failure or by degrading gas exchanges and compromising oxygen delivery in ventilated patients [3]. It is therefore essential to have reliable bedside tools to predict the efficacy of volume expansion.

Nowadays, the concept of predicting preload responsiveness rather than the traditional assessment of preload responsiveness has been widely proposed as an attractive alternative [4]. Minimal or noninvasive techniques such as transthoracic and transesophageal echocardiography (TTE and TEE, respectively) have gained wide acceptance and are routinely performed by intensivists to evaluate and monitor patients’ bedside hemodynamics [5,6]. Interestingly, both (noninvasive) TTE and (minimally invasive) TEE allow the echocardiographic examination of the superior and inferior vena cavae diameters and permit assessment of respiratory changes (respiratory variations of the superior vena cava (ΔSVC) and inferior vena cava (ΔIVC)). These cyclic changes have been proposed to reflect venous return and to serve as useful predictors of fluid responsiveness in septic patients [7-9]. Nevertheless, it is quite difficult to compare the accuracy of ΔSVC and ΔIVC, because the clinical studies that have been used to validate each parameter are not comparable in terms of patient population, fluid regimen or criteria used to define a positive response to a fluid challenge [7-9]. Physiologically, the superior and inferior vena cavae are exposed to significantly different pressures, which could explain the reported discrepancies between the predictive values of both parameters [7-9]. In fact, only 20% of the airway pressure is transmitted to the abdomen [10], and the relationship between venous transmural pressure and venous size is curvilinear [11]. One could thus expect that the increase in downstream pressure induced by mechanical insufflation may cause different diameter changes in the two vena cavae systems during mechanical ventilation. The ΔSVC and ΔIVC appear to predict fluid responsiveness equally well [7-9], even though they are exposed to different physiological mechanisms. These anatomic and physiologic differences may lead one to assume that ΔSVC is better than ΔIVC in predicting fluid responsiveness. To compare these two predictors, we prospectively studied simultaneous ΔSVC and ΔIVC recordings in a sample of mechanically ventilated septic patients in a mixed medical and surgical ICU.

## Material and methods

### Patients

This prospective study was conducted in the ICU of a university hospital (Hôpital Purpan, Toulouse, France). The study was reviewed and approved by the Institutional Review Board (Comité de Protection des Personnes Hospices Civils de Limoges, France, approval CPP10-008a/2010-A00616-33). Written informed consent was obtained from each patient’s next of kin.

Inclusion criteria were mechanically ventilated patients in septic shock (as defined by the Surviving Sepsis Campaign [1]) who required a rapid volume challenge (7 ml/kg of 6% hydroxyethylstarch for 15 minutes) as directed by the attending physician. The physician’s decision was based on the presence of clinical signs of acute circulatory failure (low blood pressure or urine output, tachycardia, mottling) and/or biological signs of organ dysfunction (renal or hepatic dysfunction, lactic acidosis), as well as on the absence of contraindication to a fluid challenge (life-threatening hypoxemia, echocardiographic evidence of right ventricular failure). Excluded were patients with spontaneous respiratory effort and/or cardiac arrhythmias, as well as those in whom an echocardiographic examination could not be performed (that is, contraindication to TEE [12] or inability to perform TTE (*n* = 4, 8%)).

### Measurements

For each patient, echocardiographic assessments were performed double-blinded simultaneously by two experienced physicians (level 3 echocardiography training) [5] using a Doppler echocardiography device (EnVisor ultrasound system; Philips, Suresnes, France) equipped with a phased array transthoracic probe (3.5 MHz) and a multiplane transesophageal probe (5 MHz). Synchronization of the measurements with the different times of the ventilatory cycle was made possible by displaying the airway pressure curve on the screen of the ultrasound system (Echo Bridge; MAQUET, Rastatt, Germany).

The IVC was examined subcostally in the longitudinal view with the transthoracic probe. Its diameter was measured using the M-mode strictly perpendicular to the vessel and immediately above the juncture with the hepatic vein. Maximal and minimal IVC diameters (Dmax_IVC_ and Dmin_IVC_, respectively) were measured over a single ventilatory cycle. The ΔIVC or the distensibility index of IVC, which reflects the increase of its diameter during mechanical insufflation, was calculated as (Dmax_IVC_ − Dmin_IVC_)/Dmin_IVC_ and expressed as a percentage [8]. A ΔIVC ≥18% has previously been shown to have the best accuracy for predicting fluid responsiveness [8], and this threshold value was tested in our patients. In addition, we tested another previously published index based on the same measurements (Dmax_IVC_ − Dmin_IVC_)/(Dmax_IVC_ + Dmin_IVC_)/2) = ΔIVC_2_), where ΔIVC_2_ ≥ 12% was the best threshold value for predicting fluid responsiveness [9].

The SVC was examined from a long-axis view with a transesophageal probe using the two-dimensional view to locate the M-mode beam across its maximal diameter, as previously described [7]. The SVC diameters (Dmax_SVC_ and Dmin_SVC_) were measured over a single respiratory cycle, and the ΔSVC or the collapsibility index of SVC was calculated as (Dmax_SVC_ − Dmin_SVC_)/Dmax_SVC_ and expressed as a percentage. As a ΔSVC >36% has been previously shown to have good accuracy for predicting fluid responsiveness [7], this threshold value was chosen for testing in our patients.

The left ventricular (LV) stroke volume was measured by using a Doppler technique with a transesophageal probe. The pulse Doppler aortic flow velocity-time integral (AoVTI) was determined at the level of the aortic annulus using a transgastric 120° view and the aortic valve area (SAo) at the level of the aortic annulus. AoVTI was measured only with TEE. The stroke volume was then calculated by multiplying AoVTI by Sao, and the cardiac index (CI) was determined by dividing the product of stroke volume and heart rate by the patient’s body surface area, as described and validated in previous studies [13]. Changes in CI before (T0) and after fluid challenge (T1) or ΔCI, were expressed as percentages. We calculated CI by using only TEE data [13].

Additionally, LV systolic function was measured before and after fluid challenge by calculating the LV fractional area change (LVFAC) as previously described [14]. A LVFAC <40% was considered as a LV dysfunction.

Measurements of Dmax_SVC_, Dmax_IVC_ and AoVTI were performed in triplicate over three consecutive respiratory cycles. The results are expressed as the mean of these three measurements. The mean interobserver and intraobserver variabilities in the measurement of Dmax_SVC_, Dmax_IVC_ and AoVTI were 8 ± 7% and 5 ± 6%, 9 ± 9% and 6 ± 8%, and 8 ± 6% and 5 ± 4%, respectively.

### Study protocol

All patients were sedated and mechanically ventilated in a volume-controlled mode with a tidal volume of 8 to 10 ml/kg. Two sets of measurements were taken. The first was prior to volume expansion, and the second was immediately after volume expansion. Ventilatory settings as well as dosages of vasopressive drugs were held constant throughout the study. All Doppler echocardiographic measurements were taken offline from videotape recordings.

### Statistical analysis

The effects of volume expansion on hemodynamic parameters were assessed using a nonparametric Wilcoxon rank-sum test. Assuming that a 15% change in CI was required for clinical significance [15,16], patients were separated into responders (R) and nonresponders (NR) on the basis of a change in cardiac output ≥15% and <15%, respectively, following the volume challenge. The comparison of hemodynamic parameters prior to volume expansion in R and NR patients was performed using a nonparametric Mann-Whitney *U* test.

Receiver operating characteristic (ROC) curves were generated for ΔSVC and ΔIVC, with the discriminating threshold varied for each parameter. The areas under the ROC curves (AUC) for ΔSVC and ΔIVC were compared using the nonparametric test published by DeLong *et al*. [17]. The sensitivity, specificity, positive predictive value and negative predictive value of ΔIVC and ΔSVC for predicting fluid responsiveness were calculated. The best cutoff of ΔIVC and ΔSVC values were defined by the best cutoff of the sensitivity and specificity of each index. Correlations between ΔSVC and ΔCI, ΔIVC and ΔCI, and ΔSVC and ΔIVC were assessed using Spearman’s ρ coefficient. Linear correlations were tested using the Spearman’s rank method. Statistical analysis was performed using R software version (2.15.1; R Project for Statistical Computing, Vienna, Austria). All *P*-values were two-sided, and a *P*-value of 0.05 was considered significant.

## Results and discussion

### Static hemodynamic approach

Forty-four patients with sepsis or septic shock were included over an 11-month period. Twenty-six patients (59%) were R. Ten patients (22.7%) died during their ICU stay. Characteristics of the study patients and comparisons between R and NR at baseline are shown in Table 1. Hemodynamic and echocardiographic data in R and NR before and after fluid challenge (T0 and T1, respectively) are shown in Table 2. At baseline (T0), CI was not significantly different between R (2.3 L ∙ min^−1^ ∙ m^−2^ (95% CI: 1.3 to 3.4)) and NR (2.4 L ∙ min^−1^ ∙ m^−2^ (95% CI: 1.3 to 3.9)) (*P* = 0.841). Overall (R and NR), heart rate, mean arterial blood pressure and central venous pressure increased significantly after volume expansion (*P* < 0.005 for all comparisons).

**Table 1:** **Characteristics of the study patients and comparison between responders and nonresponders at baseline (before fluid challenge)** ^**a**^

**Parameters**	**All patients**	**Responders**	**Nonresponders**	***P*** **-value**
	**(** ***N*** **= 44)**	**(** ***n*** **= 26)**	**(** ***n*** **= 18)**	
Age, yr	58.5 (34.8 to 82.6)	60.4 (36.0 to 84.2)	51.4 (36.7 to 71.5)	0.210
BMI	23.6 (17.3 to 35.2)	24.3 (17.6 to 40.0)	22.6 (17.7 to 28.1)	0.142
Females, *n* (%)	18 (40)	13 (30)	5 (11)	0.245
SAPS II	67.5 (36.2 to 95.8)	68.0 (39.6 to 87.3)	62.5 (34.8 to 97.3)	0.466
Norepinephrine, *n* (%)	33 (75)	20 (46)	13 (30)	1
Dose of norepinephrine, mg/h	1.7 (0.0 to 5.7)	1.3 (0.0 to 5.8)	2.0 (0.0 to 5.6)	0.91
V_t_, ml/kg	8.2 (6.4 to 11.0)	8.4 (6.5 to 11.6)	8.1 (6.1 to 9.7)	0.315
Respiratory rate, breaths/min	20 (15 to 26)	20 (15 to 26)	21 (16 to 26)	0.293
PEEP, cmH_2_O	7 (5 to 12)	7 (5 to 11)	7 (5 to 10)	0.789
Pplat, cmH_2_O	22 (15 to 27)	23 (13 to 27)	21 (16 to 27)	0.801
ARDS, *n* (%)	17 (39)	7 (27)	10 (56)	0.06
ALI, *n* (%)	12 (27)	8 (31)	4 (22)	0.105
Laparotomy, *n* (%)	10 (23)	5 (19)	5 (28)	0.76
Origin of sepsis, *n* (%)				0.88
Pulmonary	19 (43)	10 (39)	9 (50)	
Abdominal or urinary	17 (39)	10 (39)	7 (39)	
Skin	5 (11)	4 (15)	1 (6)	
Other	3 (7)	2 (8)	1 (6)	

^a^ALI, Acute lung injury (100 < PaO_2_/FiO_2_ < 300 mmHg); ARDS, Acute respiratory distress syndrome (PaO_2_/FiO_2_ < 200 mmHg); BMI: Body mass index; PaO_2_/FiO_2_, Ratio of partial pressure of oxygen in arterial blood to fraction of inspired oxygen; Pplat: Plateau pressure; SAPS II: Simplified Acute Physiology Score; V_t_, Tidal volume; PEEP, Positive end-expiratory pressure. Data are expressed as medians with fifth and ninety-fifth percentiles, unless otherwise indicated.

**Table 2:** **Hemodynamic characteristics between responders and nonresponders before and after volume expansion** ^**a**^

**Studied parameters**	**All patients**	**Responders**	**Nonresponders**	***P*** **-value**
	**(** ***N*** **= 44)**	**(** ***n*** **= 26)**	**(** ***n*** **= 18)**	
MAP, mmHg				
T0	71 (53 to 100)	73 (55 to 100)	70 (58 to 89)	0.685
T1	78 (58 to 100)	77 (68 to 102)	79 (56 to 92)	0.563
HR, beats/min				
T0	106 (68 to 141)	107 (75 to 138)	101 (61 to 142)	0.99
T1	101 (63 to 146)	102 (70 to 147)	101(61 to 142)	0.738
CVP, mmHg				
T0	10 (4 to 17)	10 (5 to 17)	8 (3 to 18)	0.326
T1	101 (63 to 146)	102 (70 to 147)	101(61 to 142)	0.738
LVFAC, %				
T0	46 (21 to 61)	50 (21 to 59)	42 (21 to 62)	0.75
T1	47 (23 to 63)	47 (25 to 63)	48 (26 to 61)	0.861
AoVTI, cm				
T0	13.7 (8.2 to 22.8)	13.0 (8.3 to 19.9)	15.6 (10.2 to 24.1)	0.06
T1	18.0 (12.0 to 23.3)	18.2 (12.0 to 23.0)	16.9 (11.2 to 23.5)	0.527
CI, L ∙ min^−1^ ∙ m^−2^				
T0	2.3 (1.3 to 3.8)	2.3 (1.3 to 3.4)	2.4 (1.2 to 4.0)	0.841
T1	2.8 (1.5 to 4.3)	3.1 (1.6 to 4.8)	2.5 (1.4 to 3.2)	0.054
Dmax_SVC,_ mm				
T0	13.2 (8.2 to 20.4)	12.0 (8.3 to 19.8)	14.0 (9.2 to 22.6)	0.05
T1	14.6 (8.9 to 21.7)	13.5 (8.5 to 22.4)	15.0 (10.4 to 19.1)	0.568
ΔSVC, %				
T0	20 (6 to 47)	31 (7 to 49)	16 (5 to 30)	0.01
T1	12 (3 to 63)	15 (4 to 68)	6 (0 to 25)	0.008
Dmax_IVC_, mm				
T0	19.6 (12.0 to 23.1)	19.0 (12.0 to 28.9)	19.9 (11.1 to 24.9)	0.67
T1	21.1 (12.9 to 28.0)	19.6 (12.1 to 28.1)	22.2 (16.1 to 25.1)	0.218
ΔIVC, %				
T0	18 (2 to 55)	12 (2 to 55)	20 (3 to 58)	0.453
T1	9 (2 to 26)	10 (0 to 30)	6 (3 to 25)	0.47

^a^AoVTI, Pulse Doppler aortic velocity time integral; CI, Cardiac index; CVP, Central venous pressure; Dmax_IVC_, Maximal diameter of inferior vena cava; Dmax_SVC_, Maximal diameter of superior vena cava; HR, Heart rate; ΔIVC, Distensibility index of inferior vena cava; LVFAC, Left ventricular fractional area change; MAP, Mean arterial pressure; ΔSVC, Collapsibility index of superior vena cava; T0, Before volume expansion; T1, After volume expansion; VTI, Velocity time integral. Data are expressed as medians with 95% confidences intervals. *P*-value corresponds to the comparison between Responders and Nonresponders at each time point (T0 and T1). Data are expressed as medians with fifth and ninety-fifth percentiles.

### Predicting fluid responsiveness

#### With superior vena cava dynamic measurements

**Figure 1:**
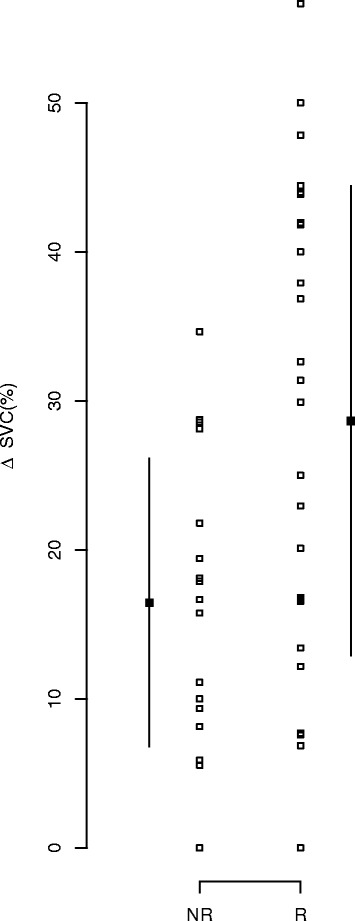
**Individual values for the superior vena cava collapsibility index according to the fluid responsiveness.** Patients were divided into two groups: responders (R) or nonresponders (NR). T0, Baseline; T1, After volume expansion. Individual values are indicated by open circles, and median ± interquartile range values are marked by closed circles. *P < 0.05 R vs. NR. ΔSVC, Collapsibility index of superior vena cava.

Individual values of ΔSVC according to fluid responsiveness are shown in Figure 1. In our sample, the best cutoff value of ΔSVC to predict fluid responsiveness was 29% with a sensitivity of 54% (95% CI: 35 to 73) and a specificity of 94% (95% CI: 83 to 105). A poor correlation between ΔSVC and ΔCI was found (*r* = 0.307, *P* = 0.04). A ΔSVC >36% allowed us to discriminate between R and NR in our sample with a sensitivity of 42% (95% CI: 23 to 61), a specificity of 100% (95% CI: 100 to 100), a positive predictive value of 100% (95% CI: 100–100) and a negative predictive value of 55% (95% CI: 38 to 72).

It is worth noting that excluding patients (*n* = 18, 40.9%) with low tidal volume (<8 ml/kg) and with low heart and respiratory rate (HR/RR) ratios (<3.6) did not significantly change the diagnostic value (sensitivity of 47% (95% CI: 23 to 71), a specificity of 100% (95% CI: 100–100), a positive predictive value of 100% (95% CI: 100–100) and a negative predictive value of 50% (95% CI: 27 to 73)).

#### With inferior vena cava dynamic measurements

**Figure 2:**
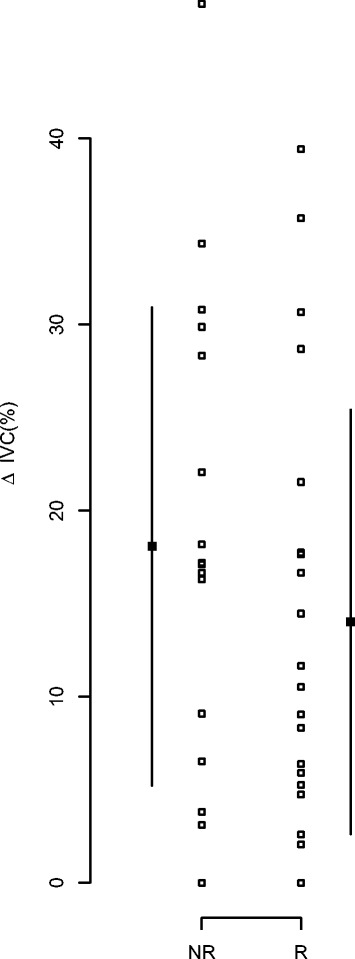
**Individual values for the inferior vena cava distensibility index according to the fluid responsiveness.** Patients were divided into two groups: responders (R) or nonresponders (NR). T0, Baseline; T1, After volume expansion. Individual values are indicated by open circles, and median ± interquartile range values are marked by closed circles. *P < 0.05 R vs. NR.

Individual values of ΔIVC according to fluid responsiveness are shown in Figure 2. In our sample, the best cutoff value of ΔIVC was 21% with a sensitivity of 38% (95% CI: 19 to 57] and a specificity of 61% (95% CI: 38 to 84). No correlation between ΔIVC and ΔCI was observed (*r* = −0.178, *P* = 0.26). In our sample, ΔIVC ≥18% allowed for discrimination between R and NR with a sensitivity of 42% (95% CI: 22 to 62), a specificity of 39% (95% CI: 16 to 62), a positive predictive value of 48% (95% CI: 27 to 69) and a negative predictive value of 33% (95% CI: 13 to 53). When patients ventilated with low tidal volume and those with low HR/RR ratios were excluded, the sensitivity was 44% (95% CI: 20 to 68) (for ΔIVC ≥18%), the specificity was 33% (95% CI: 2 to 64), the positive predictive value was 54% (95% CI: 27 to 81) and the negative predictive value was 25% (95% CI: 1 to 50). The AUC for ΔIVC_2_ (ΔIVC as described by Feissel *et al.* [9]) was similar to the ΔIVC as described by Barbier *et al.* [8] (0.43 (95% CI: 0.25 to 0.61)).

### Comparison of ΔSVC and ΔIVC as predictors of fluid responsiveness

**Figure 3:**
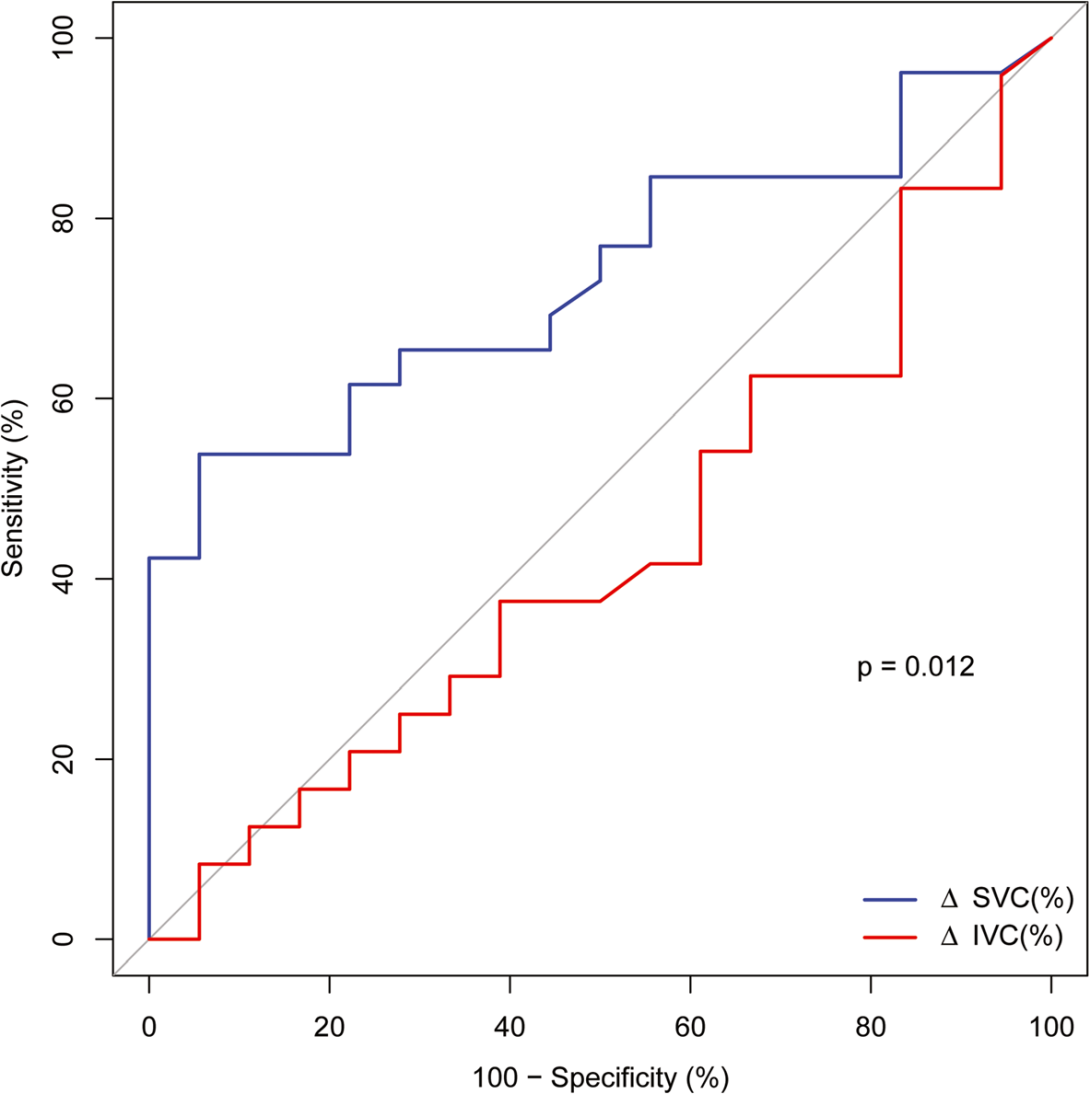
**Receiver operating characteristic curves comparing the ability of superior vena cava collapsibility index and inferior vena cava distensibility index to discriminate between responders and nonresponders.** The area under the receiver operating characteristic (ROC) curve for the collapsibility index of superior vena cava (ΔSVC) (0.74; 95% CI: 0.59 to 0.88) differed significantly from the area under the ROC curve for the distensibility index of inferior vena cava (ΔIVC) (0.43; 95% CI: 0.25 to 0.61) (*P* = 0.012).

The AUC for ΔSVC and ΔIVC regarding assessment of fluid responsiveness showed that ΔSVC showed better accuracy compared to ΔIVC (0.74 (95% CI: 0.59 to 0.88) versus 0.43 (95% CI: 0.25 to 0.61) (*P* = 0.012) (Figure 3). No significant correlation between ΔSVC and ΔIVC was found (*r* = 0.005, *P* = 0.98). ΔSVC and ΔIVC were significantly lower after volume expansion (*P* < 0.001), whereas changes for Dmax_SVC_ and Dmax_IVC_ were not significant (*P* = 0.16 and *P* = 0.06, respectively). Despite the significant difference between R and NR, the AUC of D_maxSVC_ remained low (0.67 (95% CI: 0.51 to 0.85)).

We assessed right ventricular function in all of the cases and detected three cases of right ventricular failure (right/left >0.6). In none of the three cases did we observe a difference in reactivity between IVC and SVC.

Overall, these results are in agreement with our main hypothesis of a dissociation between the ability of these dynamic vena cavae measurements to predict preload responsiveness. The better predictive value of ΔSVC could be due to a comparatively greater mechanical insufflation-induced decrease in venous return at the intrathoracic level compared to the intra-abdominal level. The greater impact of intrathoracic pressure variation could be related to (1) a greater increase in right atrial pressure (that is, in the back pressure to venous return) [10], (2) a greater increase of the right ventricular impedance due to the collapse of poorly filled alveolar vessels [18] or (3) the occurrence of a venous waterfall phenomenon between the extrathoracic and intrathoracic vena cavae segments [19]. Because our study was designed to be part of routine clinical practice, we were unable to determine which of the mechanisms described above was predominant. Furthermore, it must be highlighted that both indices were found to be less sensitive and less specific than previously reported. In our study, a ΔSVC >36% predicted fluid responsiveness with high specificity (100%) and high positive predictive value (100%), but with poor sensitivity (42%). Several explanations for such discrepancies between the present study and previously published work are possible [7,20]. First, the mechanical ventilation settings were not similar. The respiratory rate and PEEP were higher in our study than in the study by Vieillard-Baron *et al*. [7] (20 breaths/min and 5 to 11 cmH_2_O vs. 15 breaths/min and 5 to 7 cmH_2_O). The ability of the SVC to collapse in the thorax is influenced by intrathoracic pressure and volume, and different HR/RR ratios may impact its reliability to predict fluid responsiveness [21]. In our study, one-third of patients had acute respiratory distress syndrome (ARDS), and 40.9% were ventilated with low tidal volumes and had HR/RR ratios <3.6. The parameters used to assess fluid responsiveness in this patient group have been questioned [22], and the low tidal volumes required in our patients with ARDS and low pulmonary compliance may have impacted the ability of ΔSCV to predict fluid responsiveness, indicating the potential limitation of the use of this index in such patients. We mention elsewhere that, in our present study, 26 patients (60%) were ventilated with a tidal volume >8 ml/kg. Although this may seem high by today’s standards, it was, at the time of the first patient’s inclusion in March 2011, recommended to ventilate only patients with acute lung injury or ARDS with a low tidal volume (<8 ml/kg) [1]. Second, Vieillard-Baron and colleagues defined R as an increase >11% in CI, whereas we selected 15% to be consistent with data reported in the current literature [8,9,15,16,23,24]; however, the sensitivity remained poor (39%), even when we used ΔCI ≥ 11%. A larger proportion of our cohort received vasopressor support (75% versus 50%), and it has been shown that norepinephrine can affect fluid challenge [25].

Our results show that the AUC for ΔSVC regarding assessment of fluid responsiveness was low (0.74 (95% CI: 0.59 to 0.88)). Contrary to the findings of other investigators, we discovered that, in real-life ICU practice conditions, the AUC of ΔIVC seems not to be a reliable predictor of fluid responsiveness and that its TEE counterpart, ΔSVC, shows a poor fluid responsiveness except for the high variation levels.

Regarding the IVC, in contrast with previous researchers, we found a poorer sensitivity (42%) and specificity (39%), despite the fact that we used the same threshold of ΔIVC (≥18%) [8]. Several physiological hypotheses should be considered. For instance, because the IVC is mainly intra-abdominal, its ability to distend could be limited by an increase in intra-abdominal pressure, especially in postoperative abdominal surgery patients. In a recent study, the impact of intra-abdominal pressure on IVC diameter was evaluated in mechanically ventilated pigs. The results showed that IVC diameters are affected by intra-abdominal pressure and that fluid responsiveness should not be estimated from retrohepatic IVC diameter in cases of high intra-abdominal pressure [26]. Our results show a weak AUC for ΔIVC (0.43 (95% CI: 0.25 to 0.61)), suggesting that ΔIVC may not be a consistently reliable predictor of fluid responsiveness.

Our results, as compared to those of previously published studies, suggest that in ARDS patients, a standard ventilation strategy, high vasopressor infusion rate and/or abdominal surgery may alter the ability of ΔSVC and ΔIVC to predict fluid responsiveness and that these predictive indices should be investigated extensively and refined before any generalized use can be recommended. Furthermore, the selection of a single cutoff point for making clinical decisions may be too simplistic. A “gray zone” approach applied to the pulse pressure variations for prediction of fluid response in mechanically ventilated patients under general anesthesia was recently suggested by Cannesson *et al.* [27]. This “gray zone” approach has not been used in our study, because the size of our cohort did not permit such statistical analysis.

## Conclusions

In a heterogeneous sample of mechanically ventilated septic patients in medical and surgical ICUs, ΔSVC appeared to have better accuracy than ΔIVC for predicting fluid responsiveness. A cutoff >36% identified R with high specificity and positive predictive value. However, our results also suggest that the accuracy of both ΔSVC and ΔIVC as predictors of fluid responsiveness is lower than that reported in the literature, thus raising questions about their reliability in patients with ARDS, postoperative abdominal surgery patients or patients treated with high vasopressor infusion rates. In our opinion, a complete evaluation of volume status in septic and mechanically ventilated patients should include both IVC and SVC examinations.

## Key messages

- ΔSVC appears to have better accuracy than ΔIVC for predicting fluid responsiveness in ventilated septic patients.
- The accuracy of both ΔSVC and ΔIVC as predictors of fluid responsiveness was lower than data reported in the literature, raising questions about their reliability in patients with ARDS, postoperative abdominal surgery patients or patients treated with high vasopressor infusion rates.

## Abbreviations

AoVTI: Pulse Doppler aortic velocity time integral
ARDS: Acute respiratory distress syndrome
BMI: Body mass index
CI: Cardiac index
CVP: Central venous pressure
Dmax_IVC_: Maximal diameter of inferior vena cava
Dmax_SVC_: Maximal diameter of superior vena cava
HR: Heart rate
HR/RR: Heart rate/respiratory rate
LVFAC: Left ventricular fractional area change
MAP: Mean arterial pressure
NR: Nonresponders to fluid challenge
PEEP: Positive end-expiratory pressure
Pplat: Plateau pressure
R: Responders to fluid challenge
SAPS II: Simplified Acute Physiology Score
V_t_: Tidal volume
ΔIVC: Distensibility index of inferior vena cava
ΔSVC: Collapsibility index of superior vena cava
